# Large Intron Inversions in Romanian Patients with Hemophilia A—First Report

**DOI:** 10.3390/medicina59101821

**Published:** 2023-10-13

**Authors:** Melen Brinza, Andra Grigore, Mihaela Dragomir, Dumitru Jardan, Cerasela Jardan, Paul Balanescu, Claudia Cristina Tarniceriu, Oana Viola Badulescu, Cristina Blag, Ciprian Tomuleasa, Adina Traila, Margit Serban, Daniel Coriu

**Affiliations:** 1 Department of Hematology and Bone Marrow Transplant, Fundeni Clinical Institute, 022328 Bucharest, Romania; 2 Department of Hematology, “Carol Davila” University of Medicine and Pharmacy, 020021 Bucharest, Romania; 3 Molecular Biology Laboratory, Medlife, 010093 Bucharest, Romania; 4 Internal Medicine Chair, “Carol Davila” University of Medicine and Pharmacy, 020021 Bucharest, Romania; 5 Department of Anatomy, “Grigore T. Popa” University of Medicine and Pharmacy, 700115 Iasi, Romania; 6 Department of Hematology, “St Spiridon” County Clinical Emergency Hospital, 700111 Iasi, Romania; 7 Department of Pathophysiology, “Grigore T. Popa” University of Medicine and Pharmacy, 700115 Iasi, Romania; 8 Pediatric Discipline, “Iuliu Hatieganu” University of Medicine and Pharmacy, 400177 Cluj Napoca, Romania; 9 Pediatric Clinic, Emergency Clinical Hospital for Children, 400177 Cluj Napoca, Romania; 10 Department of Hematology, “Iuliu Hatieganu” University of Medicine and Pharmacy, 400012 Cluj Napoca, Romania; 11 Department of Hematology, “Ion Chiricuta” Clinical Cancer Center, 400124 Cluj Napoca, Romania; 12 “Cristian Serban” Medical Center for Evaluation Therapy, Medical Education and Rehabilitation of Children and Young Adults, European Hemophilia Treatment Centre, 305100 Buzias, Romania; 13 Department of Onco-Hematology, “Louis Turcanu” Emergency Hospital for Children, 300011 Timisoara, Romania; 14 Department of Hematology, “Victor Babes” University of Medicine and Pharmacy, 300041 Timisoara, Romania

**Keywords:** hemophilia A, intron 22 inversion, intron 1 inversion, Romania, Eastern Europe

## Abstract

*Background and Objectives*: Despite the vast heterogeneity in the genetic defects causing hemophilia A (HA), large intron inversions represent a major cause of disease, accounting for almost half of the cases of severe HA worldwide. We investigated the intron 22 and intron 1 inversion status in a cohort of Romanian unrelated patients with severe HA. Moreover, we evaluated the role of these inversions as relative risk factors in inhibitor occurrence. *Materials and Methods*: Inverse shifting—a polymerase chain reaction method was used to detect the presence of intron 22 and intron 1 inversions in 156 Romanian patients with HA. *Results*: Intron inversion 22 was found in 41.7% of the patients, while intron 1 inversion was detected in 3.2% of the patients. Overall, large intron inversions represented the molecular defect in 44.9% of the studied patients. Our findings are in accord with previously published reports from Eastern Europe countries and with other international studies. The risk of inhibitor development was higher in patients with inversion 1 compared to the patients with HA without any inversion detected. *Conclusions*: The current study demonstrates the major causative role of large intron inversions in severe HA in Romanian patients. Moreover, our study confirms the contribution of intron 1 inversion in inhibitor development.

## 1. Introduction

Hemophilia A (HA) is a common congenital X-linked bleeding disorder defined by a deficiency in plasma coagulation factor VIII (FVIII). The incidence of HA varies between 1/5000 and 1/10,000 live male births [1]. The severity of the disease correlates with the level of residual coagulation activity of plasma FVIII:FVIII, < 1% in severe HA (50% of cases), between 2–5% in moderate HA (10% of cases) and between 5–30% in mild HA (30–40% of cases) [2].

FVIII is encoded by the F8 gene, located distally on the long arm of the X chromosome (Xq28). The F8 gene spans 186 kb and consists of 26 exons and 25 introns, being one of the largest genes in the genome [3]. Several introns of the F8 gene have an extreme length of over 14 kilobases (kb) [4]. Among them, intron 1 and intron 22 are of particular interest regarding the etiopathology of severe HA. Intron 1 contains a 1041-bp region designated as intron 1 homologous region (int1h-1). A region highly homologous to int1h-1 is situated outside the F8 gene, upstream of exon 1 (int1h-2). Intrachromosomal homologous recombination between int1h-1 and int1h-2 produces an inversion reported as the cause of 1–5% of all severe forms of HA [5,6]. Intron 22 also contains a homologous region of 9.5 kb, named int22h-1. Outside the F8 gene, towards the telomere, there are two regions highly homologous to int22h-1: int22h-2 and int22h-3. Intrachromosomal homologous recombination can occur either between int22h-1 and int22h-2 or between int22h-1 and int22h-3, leading to proximal/type II inversion and distal/type I inversion, respectively. Together, the two types of inversion 22 account for 40–45% of the cases of severe HA worldwide [7,8].

Although HA is genetically heterogeneous, with more than 3000 distinct mutations reported up-to-date, approximately half the cases of severe disease are produced by large intron inversions [9].

The molecular diagnosis of large intron inversions from HA has evolved over time from the labor-intensive Southern Blot designed by Lakich et al. in 1993 [10] to time-consuming long distance-polymerase chain reaction (LD-PCR)-based methods by Liu et al. in 1998 [11], to simpler and more rapid inverse shifting-PCR (IS-PCR) by Rossetti et al. in 2005 [12] and to newer protocols based either on LD-PCR or IS-PCR that enable the identification of the type of int22h rearrangement [13,14]. One of the newer protocols based on LD-PCR followed by nested PCR was published recently by Wang et al., providing an alternative for the detection of inversion 22 [15].

We aimed to study, for the first time, the prevalence of inversion 22 and inversion 1 in Romanian patients with severe HA by means of IS-PCR and to compare our results with data previously reported in Eastern Europe populations. Another objective is to assess the role of large intron inversions as risk factors for inhibitor development in our cohort.

## 2. Materials and Methods

Our study was cross-sectional and included 156 apparently unrelated patients previously diagnosed with severe HA who attended successively one of the following National Centers of Hemophilia: Fundeni Clinical Institute from Bucharest, “Sf. Spiridon” Clinical Emergency Hospital from Iasi, “Prof. Dr. Ion Chiricuta” Institute of Oncology from Cluj-Napoca and “Cristian Serban” Medical Center for Evaluation and Rehabilitation for Children from Buzias. We excluded from the study group related patients and also patients with mild and moderate forms of HA. Of the 156 patients, 44.9% were sporadic cases, 48.1% belonged to families with history of HA, while family history was unknown in 7.9% of the participants in the study.

The group of patients was heterogeneous regarding age and therapeutic regimen. The mean age of the patients was 40 years (SD = 14). One-third of the patients (33.3%) received on demand treatment, while 66.7% of them underwent prophylactic treatment. Patients were tested for inhibitors systematically according to the national protocol for HA. Inhibitors were detected previously in 14 patients.

Peripheral whole blood was collected in EDTA tubes and genomic DNA was extracted by the salting-out method followed by ethanol precipitation (PureLink Genomic DNA Mini Kit, Invitrogen, Waltham, MA, USA). DNA purity and concentration was assessed using NanoDrop-1000 spectrophotometer (NanoDrop Technologies, Wilmington, DE, USA).

The IS-PCR protocol was based on the method described by Rossetti et al. [13]. We adapted and optimized the 3-step protocol. A quantity of 500 ng of genomic DNA was digested with fast-acting BclI restriction enzyme (FastDigest BclI, Thermo Fischer Scientific, Vilnius, Lithuania) for 5 min at 37 °C, followed by enzyme inactivation for 20 min at 80 °C. Circular DNA was obtained by self-ligation with rapid acting T4 DNA ligase (Rapid DNA Ligation Kit, Thermo Fischer Scientific, Vilnius, Lithuania) at 22 °C for 5 min in a 50 µL volume. For each patient, we performed 3 polymerase chain reactions using circular DNA: two reactions for inversion 22 (diagnosis and complementary test) and one reaction for inversion 1. The primer sequences are displayed in Supplementary Materials.

Each PCR reaction was performed in a volume of 25 µL and included 3 µL DNA, 0.4 µL of the appropriate primers and 12.5 µL HotStarTaq Polymerase (Qiagen, Germany). The initial denaturation (15 min at 95 °C) was followed by 35 cycles of denaturation (30 s at 94 °C), annealing (30 s at 57 °C) and extension (1 min at 72 °C). Th final extension was performed in 10 min at 72 °C. Amplification products were detected by means of electrophoresis on 2% agarose gel stained with ethidium bromide and photographed under ultraviolet illumination.

Although the IS-PCR protocol was previously validated [13], several samples were analyzed also at the Department of Medical Genetics of the University Medical Center Utrecht, The Netherlands.

Data analysis was performed using IBM SPSS Statistics software (version 27) for Mac OS (IBM Corp., Armonk, NY, USA). We used Fisher’s exact test to compare the frequencies of inversion 22 and inversion 1 in Romanian patients with other populations from Eastern Europe. *p* values lower than 0.05 were considered statistically significant. Differences in inhibitor occurrence were evaluated using Pearson’s chi-square test. The odds ratio was calculated and interpreted as a relative inhibitor risk.

## 3. Results

**Figure 1:**
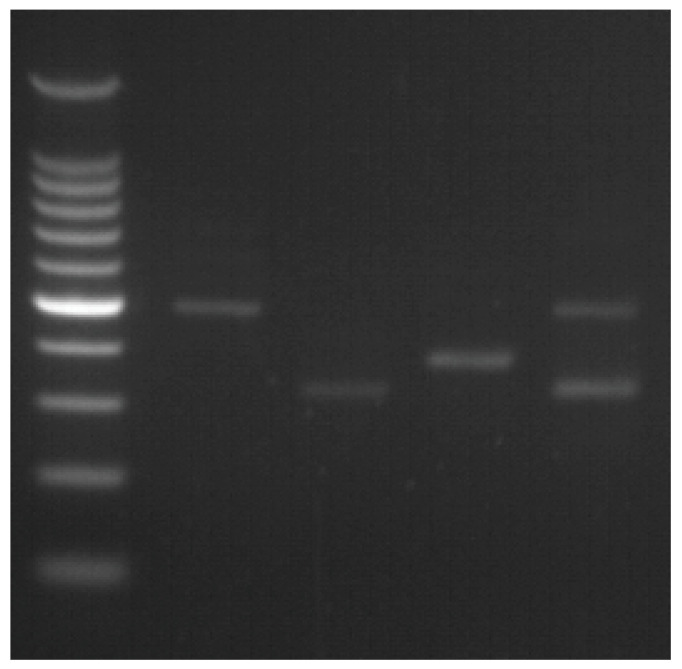
Inversion 22 diagnostic test. (1) inversion 22 negative; (2) inversion 22 type I; (3) inversion 22 type II; (4) inversion 22 type I carrier.

The amplicon length in agarose gel electrophoresis in the inversion 22 diagnostic test was 487 bp for the normal allele, 333 bp for patients with inversion 22 type I and 385 bp for those with inversion 22 type II (Figure 1).

**Figure 2:**
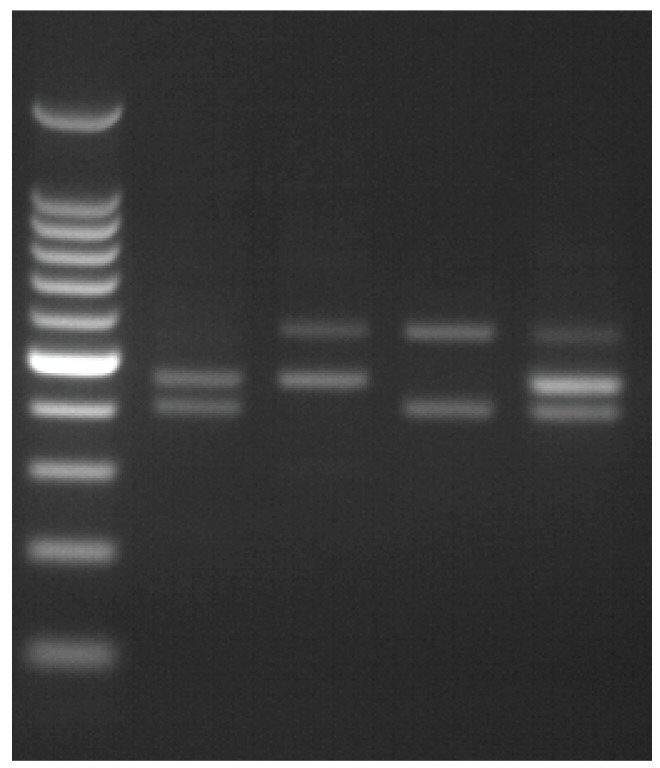
Inversion 22 complementary test. (1) inversion 22 negative; (2) inversion 22 type I; (3) inversion 22 type II; (4) inversion 22 type I carrier.

In the complementary test, the normal allele displayed two bands of 405 bp and 457 bp, respectively, while the inversion 22 type I was associated with 457 bp and 559 bp bands and inversion 22 type II was associated with 405 bp and 559 bp bands (Figure 2).

Amplicon bands with different patterns from those mentioned above were not identified in our study group.

**Figure 3:**
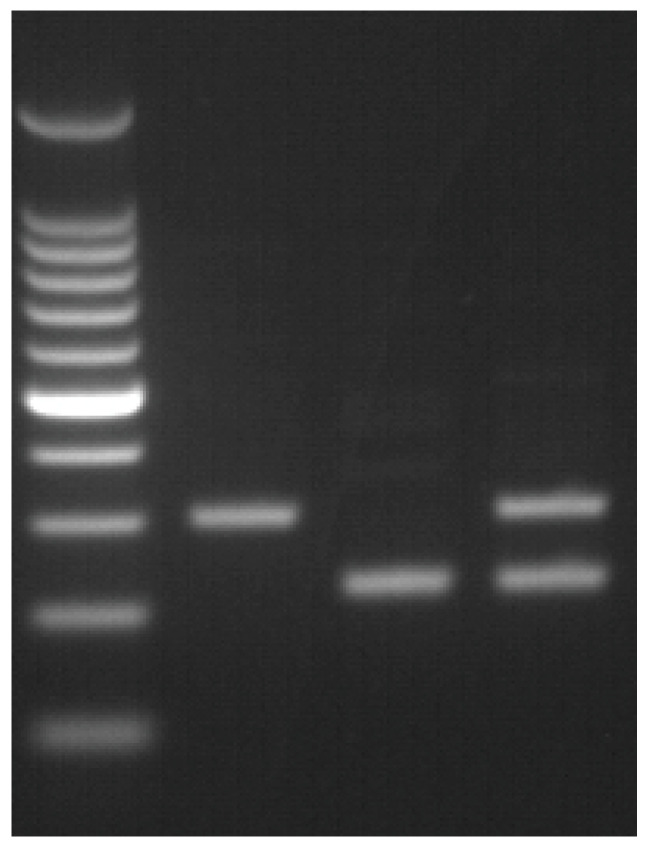
Inversion 1 diagnostic test. (1) inversion 1 negative; (2) inversion 1; (3) inversion 1 carrier.

Agarose gel electrophoresis of inversion 1 PCR amplicon revealed a 304 bp band for the normal allele and a 224 bp band for inversion 1 associated allele (Figure 3).

We identified the presence of inversion 22 in 65 out of 156 unrelated patients with severe HA (41.7%); 56 patients displayed type I inversion 22 (35.9%) and 9 patients (5.8%) exhibited a type II inversion 22 pattern. Inversion 22 type I was more frequent, being responsible for 86.2% of the cases, while type II was present only in 13.8% of cases. Intron 1 inversion was detected as the underlying genetic defect in 5 patients (3.2%) out of 156. In 86 patients (55.1%), no large intron inversion was revealed.

Inhibitors were present in seven patients with distal inversion 22 (12.5%), in two patients with inversion 1 (40%) and in five patients (5.8%) negative for large intron inversions. Overall, nine percent of the patients from the study group developed inhibitors. The difference in inhibitor occurrence between patients with inversion 1 and patients without any inversion was significant statistically, the former having 10.8-fold increased risk than the latter (the odds ratio was 10.80, with 95% confidence interval 1.45–80.14). The mean age of patients with inhibitors was 40 years (SD = 15), not significantly different from the whole study group. All patients with inhibitors were on previous on-demand treatment with plasma derived FVIII concentrates, with few exposure days. In four cases, inhibitor development was triggered by the administration of high doses of FVIII concentrates in the context of severe bleedings, and in one case, inhibitor appearance followed the substitutive treatment with high doses of FVIII after surgery in a patient with viral hepatitis C infection. Two patients had positive family history for inhibitors.

## 4. Discussion

Altogether, large intron inversions account for 44.9% of the molecular defects causative of severe HA. Our results are consistent with previous findings such as the international consortium study of Antonarakis et al., which involved over 2000 unrelated patients with severe HA and revealed a frequency of 42% of the intron 22 inversion [8]. In the same study, the type I inversion 22 was 5.5 times more frequent than type II [8], while in our study, type I inversion was approximately 6 times more frequent than type II. The cause for the higher frequency of inversion 22 type I compared with type II remains still unknown. The frequency of inversion 1 varies, usually between 0% and 5% in different reports. Summing data from over 4000 hemophilic patients from different countries, Faridi et al. reported a worldwide mean of 2.6% for the inversion in intron 1 [16]. No significant difference was detected between these data and our findings.

In addition, we reviewed 14 studies carried out in 11 Eastern European countries involving 1060 unrelated patients with severe HA. The results of these studies and the comparison with our data are summarized in Table 1.

The reported frequency for inversion 22 in Eastern Europe spans from 38% in the Russian Federation to 58.8% in a small cohort from Hungary. Type I inversion 22 was found in 80–87.5% of cases, while type II was detected in 12.5–20% of the patients with inversion 22. Our results do not differ significantly from the reviewed studies, except for one report by Bors et al. [22] from Hungary (*p* = 0.01). Inversion 1 was less studied, being reported only in nine studies with a frequency between 0% (in Poland, Bulgaria and Croatia) and 7.3% (in Macedonia). However, the majority of cohorts were small, including less than 70 patients. Our findings were similar to the reviewed data, with no significant difference being observed.

Gathering the data from the reviewed studies with our current results, we found a cumulative frequency of inversion 22 and inversion 1 in Eastern Europe populations of 46.4% (38–58.8%) and 2.9% (0–7.3%), respectively. The total number of patients with severe HA studied was 1216 for inversion 22 and 800 for inversion 1.

Even though the current study does not bring new findings regarding the prevalence of large intron inversions in severe HA, our substantial cohort of 156 patients with severe HA increases the value of collected data and provides greater statistical relevance.

Although the pathophysiological mechanism involved in inhibitor development in patients with HA is complex and not completely understood, the underlying genetic defect plays an important role [30]. The highest risk of inhibitor development is associated with large deletions and nonsense mutations in the light chain, while inversions in intron 22 and intron 1 carry an intermediate risk with an inhibitor prevalence of 21% and 17%, respectively [31]. In our study group, the prevalence of inhibitors was highest for patients with inversion 1, but this finding needs confirmation on a larger number of patients with inversion 1.

The environmental factors associated with inhibitor occurrence are positive family history (this factor could be explained by the presence of the same causative mutation), the intensity of treatment and the use of recombinant FVIII. Inhibitor development was reported also in patients with severe bleedings, surgery, concomitant infections and vaccination [30]. Except for the use of recombinant FVIII and vaccination, environmental factors played important roles in inhibitor appearance in our study.

However, the presence of inhibitors in our study (9%) was significantly lower than the incidence reported previously in patients with severe HA (approximately 30%) [32]. One reason that could explain this difference could be the genetic background of Romanian patients included in the study. Identification of the causative mutations in patients with severe HA, negative for large intron inversions and the evaluation of the interactions between genetic factors and environmental factors involved in inhibitor occurrence represent important areas for further investigation. Moreover, knowing the causative genetic defect from diagnosis/early life could majorly impact the therapeutic decision in patients with high-risk mutations (e.g., choosing regular prophylaxis over on-demand treatment and avoiding exposure to high doses of factor VIII).

## 5. Conclusions

As expected, our study confirms, for the first time, that large intron inversions represent a major cause of severe HA in Romanian patients. Our findings are similar to other Eastern European and worldwide reports. In our cohort, the presence of inversion 1 was associated with a higher risk of inhibitor development compared with patients without any large intron inversion. The IS-PCR approach is robust and cost-effective, making possible not only carrier identification and prenatal diagnosis but also the risk prediction for inhibitor development.

## Figures and Tables

**Table 1:** Frequency of intron 22 and intron 1 inversions in patients with severe HA from Eastern Europe.

Country	Number of Patients Tested for Inv22	Inv22	Inv22 I	Inv22 II	Number of Patients Tested for Inv1	Inv1	Reference
Bulgaria	40	19 (47.5%)	NA *	NA	40	0 (0%)	[17]
Croatia	68	28 (41.2%)	NA	NA	68	0 (0%)	[18]
Czech Republic	162	71 (44%)	NA	NA	162	7 (4.3%)	[19]
Hungary	104	54 (52%)	43 (80%)	11 (20%)	NA	NA	[20]
Hungary	34	20 (58.8%)	16 (80%)	3 (15%)	NA	NA	[21]
Hungary	145	82 (57%)	NA	NA	63	2 (3%)	[22]
Macedonia	55	23 (41.8%)	NA	NA	55	4 (7.3%)	[17]
Moldova	33	13 (39%)	NA	NA	33	1 (3%)	[23]
Poland	113	57 (50.5%)	47 (82.5%)	10 (17.5%)	NA	NA	[24]
Poland	56	29 (52%)	25 (86%)	4 (14%)	56	0 (0%)	[25]
Romania	156	65 (41.7%)	56 (86.2%)	9 (13.8%)	156	5 (3.2%)	Current study
Russian Federation	117	45 (38%)	NA	NA	117	1 (<1%)	[26]
Serbia	50	21 (42%)	17 (81%)	4 (19%)	50	3 (6%)	[27]
Slovakia	44	22 (50%)	18 (81.8%)	4 (18.2%)	NA	NA	[28]
Slovenia	39	16 (41%)	14 (87.5%)	2 (12.5%)	NA	NA	[29]
Total	1216	565 (46.4%)	236 (83.4%)	47 (16.6%)	800	23 (2.9%)	

* NA, not available.

## Data Availability

Data used in this study may be provided by the corresponding author upon reasonable request.

## Supplementary Materials

The following supporting information can be downloaded at: https://www.mdpi.com/article/10.3390/medicina59101821/s1, Table S1: Sequence of IS-PCR primers.
