# Supplementary material for: Large Intron Inversions in Romanian Patients with Hemophilia A—First Report
Source: Medicina (Kaunas). 2023 Oct 13;59(10):1821. doi: 10.3390/medicina59101821 (PMC10608589; doi:10.3390/medicina59101821)
Supplement: Supplementary file 1 [file medicina-59-01821-s001.zip › medicina-2635052-supplementary.pdf]

Table S1. Sequence of IS-PCR primers

	Primer	Sequence (5' to 3')	Reference
Primers for inversion 22 - diagnostic test	IU	CCTTTCAACTCCATCTCCAT	[10]
	2U	ACGTGTCTTTTGGAGAAGTC	[13]
	3U	CTCACATTGTGTTCTTGTAGTC	[13]
	ID	ACATACGGTTTAGTCACAAGT	[10]
Primers for inversion 22 - complementary test	IU	CCTTTCAACTCCATCTCCAT	[10]
	2U	ACGTGTCTTTTGGAGAAGTC	[13]
	3U	CTCACATTGTGTTCTTGTAGTC	[13]
	ED	TCCAGTCACTTAGGCTCAG	[10]
Primers for inversion 1	1-IU	GCCGATTGCTTATTTATATC	[13]
	1-ID	TCTGCAACTGGTACTCATC	[13]
	1-ED	GCCTTTACAATCCAACACT	[13]
